# Total neoadjuvant therapy improves survival of patients with borderline resectable pancreatic cancer with arterial involvement

**DOI:** 10.1002/ags3.12726

**Published:** 2023-08-10

**Authors:** Takahiro Akahori, Taichi Terai, Minako Nagai, Kota Nakamura, Yuichiro Kohara, Satoshi Yasuda, Yasuko Matsuo, Shunsuke Doi, Takeshi Sakata, Masayuki Sho

**Affiliations:** ^1^ Department of Surgery Nara Medical University Nara Japan

**Keywords:** antineoplastic combined chemotherapy protocols, chemoradiotherapy, neoadjuvant therapy, pancreatic cancer, retrospective studies

## Abstract

**Aim:**

This study aimed to evaluate the prognostic impact of total neoadjuvant therapy (TNT) for borderline resectable pancreatic cancer with arterial involvement (BR‐A) pancreatic cancer.

**Methods:**

We analyzed 81 patients initially diagnosed as BR‐A who received initial treatments between 2007 and 2021. Among them, 18 patients who received upfront surgery were classified as the UFS group, while 30 patients who were treated with neoadjuvant chemoradiotherapy were classified as the NACRT group. Furthermore, 33 patients who planned to receive a combination treatment of over 6 months of systemic chemotherapies followed by chemoradiotherapy before surgery were classified as the TNT group.

**Results:**

There were no significant differences in the patients’ backgrounds between the three groups at the time of initial treatment. The resection rates of the UFS, NACRT, and TNT groups were 89%, 77%, and 67%, respectively. NACRT had no impact on the prognosis compared to upfront surgery. In sharp contrast, the TNT group had a significantly better prognosis compared to the other groups, especially after pancreatic resection. Multivariate analysis demonstrated that TNT and resection were independent prognostic factors for the patients of BR‐A.

**Conclusion:**

TNT can be a promising therapeutic strategy for patients with BR‐A.

## Graphical abstract

The total neoadjuvant strategy for patients with borderline resectable pancreatic cancer with arterial involvement improved the prognosis compared with upfront surgery or neoadjuvant chemoradiotherapy. Total neoadjuvant therapy can be a promising therapeutic strategy.

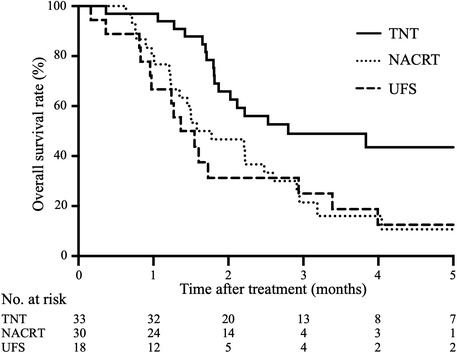

## 1 INTRODUCTION

The National Comprehensive Cancer Network (NCCN) guideline defines pancreatic cancer as resectable, borderline resectable, and unresectable tumors based on diagnostic imaging studies.^1^ Borderline resectable pancreatic cancer (BRPC) is further classified as the subset of pancreatic cancer which involves either vein (BR‐PV) or artery (BR‐A).^1^ As BRPC is considered an intermediate state between resectable and unresectable disease, it may pose not only an anatomical difficulty for surgery but also potential occult metastasis at diagnosis.^2^ Therefore, the patients with BRPC are at high risk for a margin‐positive resection and for relapse at an early period after curative intent pancreatectomy. Recent studies indicate that upfront surgery offers only minimal survival benefits for patients with BRPC.^2^, ^3^, ^4^ Therefore, despite the limited evidence to support neoadjuvant therapy, it is recommended for BRPC by most treatment guidelines. Several studies have evaluated the efficacy of neoadjuvant therapy consisting of chemotherapy or chemoradiotherapy (CRT) to improve overall survival in patients with BRPC.^5^, ^6^, ^7^ However, short‐term preoperative treatment with chemotherapy or CRT alone is challenging to provide a sufficient prognostic impact.^2^, ^7^, ^8^ We have previously reported the outcomes of the neoadjuvant chemoradiotherapy (NACRT) using gemcitabine (GEM) and concomitant radiation of 54 Gy according to each resectability status.^9^ As a result, concerning resectable and BR‐PV pancreatic cancer, the patients treated with NACRT had a significantly better prognosis than patients without NACRT. In sharp contrast, there was no significant impact on the prognosis of patients with BR‐A pancreatic cancer. Therefore, there is an urgent need to establish any powerful therapeutic strategies other than short‐term neoadjuvant therapy to improve the prognostic outcomes of BR‐A.

The concept of total neoadjuvant therapy (TNT), in which usually upfront systemic chemotherapy is followed by preoperative chemoradiotherapy in the neoadjuvant setting, has been introduced and widely accepted with favorable results, especially for rectal cancer.^10^, ^11^ Although the definition of TNT has not yet been precisely established in pancreatic cancer, to effectively manage both positive margin risk and occult metastases, some institutions have employed TNT for both BRPC and unresectable locally advanced pancreatic cancer.^12^, ^13^, ^14^ These demonstrated that TNT may have a high impact on pathological effects and prognosis. However, the published data are still insufficient for performing universally for BRPC. Furthermore, the optimal indication, treatment period, and prognostic impact of TNT, specifically for one subtype of BRPC, remain undetermined.

Based on our data, to improve the prognosis of patients with BR‐A pancreatic cancer, we initiated TNT since 2014. This novel study aimed to determine an optimal treatment strategy for BR‐A pancreatic cancer. Therefore, we retrospectively evaluated the efficacy of TNT in patients with BR‐A by comparing data from upfront surgery (UFS) and conventional NACRT.

## 2 METHODS

### 2.1 Patients

After obtaining our institutional review board approval (registration number: 2383), we reviewed a prospectively maintained database of patients with pancreatic cancer who started treatment between January 2007 and June 2021 at the Nara Medical University Hospital.

A total of 81 patients who were diagnosed with BR‐A pancreatic cancer without distant metastases were enrolled in this study. All patients were histologically or cytologically diagnosed before initiating anticancer treatment. The patients were evaluated using multidetector computed tomography and magnetic resonance imaging before treatment. Based on this imaging study, the resectability status was classified according to the NCCN Guidelines.^1^ When we suspected distant metastasis by imaging, we selectively performed staging laparoscopy to confirm negative cytology and no occult metastasis. If we found distant metastases or positive cytology, we defined the patients as unresectable and treated them with systemic chemotherapy. In this study, we did not include those patients. Patients underwent pancreatectomy, including pancreatoduodenectomy, distal pancreatectomy, distal pancreatectomy with en bloc celiac axis resection, and total pancreatectomy, if distant metastases were not detected during laparotomy or by imaging re‐evaluation before surgery or during the period of neoadjuvant treatment.

### 2.2 Neoadjuvant and adjuvant treatment

The NACRT regimen consisted of GEM and concomitant radiation at 54 Gy, as previously reported.^6^, ^9^ Systemic GEM (1000 mg/m^2^) was administered weekly. Radiotherapy was delivered through 5–9 fields for a single course of 54 Gy in 27 fractions using an intensity‐modulated radiation technique with a 6‐megavoltage photon beam (Novalis Shaped Beam Surgery System, BRAINLAB). Surgery was performed within 3–5 weeks after the completion of NACRT.

**FIGURE 1:**
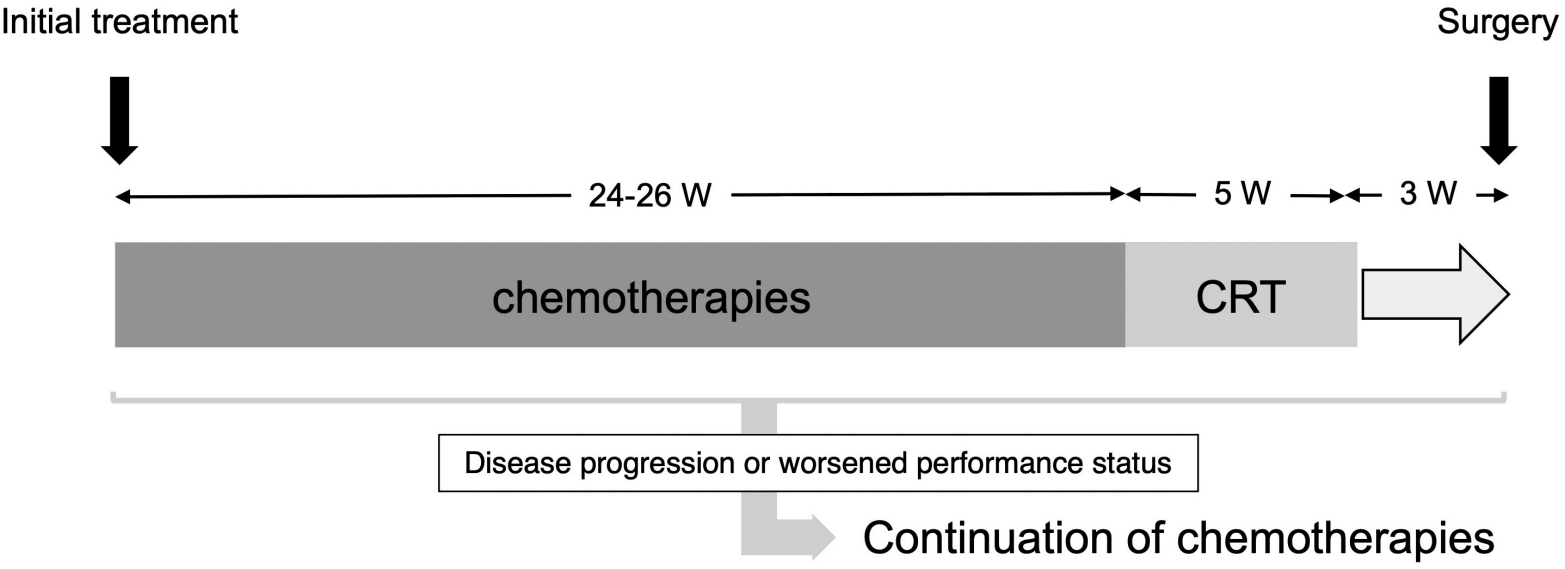
Diagram of total neoadjuvant therapy (TNT). Patients are started treatment mainly with mFOLFIRINOX (SOXIRI) or gemcitabine‐nab paclitaxel at the discretion of the treating physicians. After 24–26 weeks (W) of systemic treatment of chemotherapies, chemoradiotherapy (CRT) was performed in about 5 W. The patients were followed with radiological imaging studies every 2–3 months and laboratory studies every month throughout the neoadjuvant period. If the distance metastases appeared during TNT, the resection was not done and systemic chemotherapies were continued.

Since 2014, we used TNT to treat BR‐A pancreatic cancer. TNT includes the combination treatment with systemic chemotherapies followed by CRT for at least 8 months before surgery (Figure 1). Three regimens, including modified FOLFIRINOX (i.e., SOXIRI), gemcitabine/nab‐paclitaxel (GA), and gemcitabine plus S‐1 (GS), were mainly used for chemotherapy for at least 6 months before CRT. The SOXIRI regimen included 80 mg/m^2^ of S‐1 twice daily for 2 weeks in alternate‐day administration, 150 mg/m^2^ of irinotecan on day 1, and 85 mg/m^2^ of oxaliplatin on day 1 of a 2‐week cycle, as we previously reported.^15^ GA and GS were administered according to the standard protocol as previously reported.^16^, ^17^ The choice of the regimen for the first‐line chemotherapy or period of switching over to the other regimens was determined at the discretion of the physicians.

We usually used upfront systemic chemotherapies for at least 6 months. When we confirmed that the tumor was technically resectable by imaging, we then considered employing CRT. We usually performed surgery when we confirmed that the tumor was resectable and tumor markers were not elevated. We did not set strict criteria for tumor markers for surgery. However, if tumor markers were elevated after systemic chemotherapy or CRT, we carefully reevaluated the indication of surgery. If the tumor was technically unresectable or the tumor marker was elevated, systemic chemotherapy was usually continued, and the timing of transitioning to CRT was reevaluated every 2–3 months.

For the adjuvant therapy, some patients received combination therapy comprising a weekly hepatic arterial chemotherapy infusion of weekly high‐dose 5‐fluorouracil (WHF) combined with a systemic infusion of GEM based on prior reports.^18^ Other patients received adjuvant chemotherapy with GEM and/or S‐1 (TS‐1; Taiho Pharmaceutical) based on the condition or choice of the patients. Regardless of the dosing period, the completion of adjuvant therapy was defined, when the planned number or cycles of chemotherapy were reached: WHF/GEM, a total of nine times infusions of WHF and 18 times administrations of GEM; GEM, 18 times administrations of GEM; and S‐1, 16 weeks of oral administration.

### 2.3 Data collection

Patient demographics, surgical treatments, and postoperative outcomes were obtained through a standardized retrospective review of the electronic database of our hospital. The body mass index was calculated as weight (kg)/height^2^ (m^2^). Onodera's prognostic nutritional index (PNI) was used as a barometer of nutritional assessment. PNI was calculated as 10 × albumin (g/dL) + 0.005 × total lymphocyte count (per mm^3^).^19^ Stage classification for the evaluation of resected specimens was performed according to the eighth edition of the American Joint Committee on Cancer/Union for International Cancer Control tumor (T), node (N), metastasis classification. R0 resection was defined as 0 mm‐rule. Postoperative complications were graded according to Clavien–Dindo classifications.^20^ Pancreatic fistula was defined based on the guidelines of the International Study Group on Pancreatic Fistula.^21^ The overall survival (OS) and recurrence‐free survival (RFS) were calculated until the last follow‐up or death.

### 2.4 Statistical analysis

The clinicopathological parameters were compared using the chi‐square test, whereas continuous variables were compared using analysis of variance or the Kruskal–Wallis test, as appropriate. Continuous variables were expressed as median (range). Survival rates were calculated using the Kaplan–Meier method, and statistical significance was determined using the log‐rank test. A Cox proportional hazards model was used for univariate and multivariate analyses of the prognosis. Factors with a *p*‐value < 0.05 on univariate analysis were entered into multivariate analysis with a Cox proportional hazards model to identify independent prognostic factors. Statistical significance was set at *p* < 0.05. All statistical analyses were performed using the JMP statistical discovery software (JMP version 14.2.0, SAS Institute).

## 3 RESULTS

### 3.1 Patient characteristics

**FIGURE 2:**
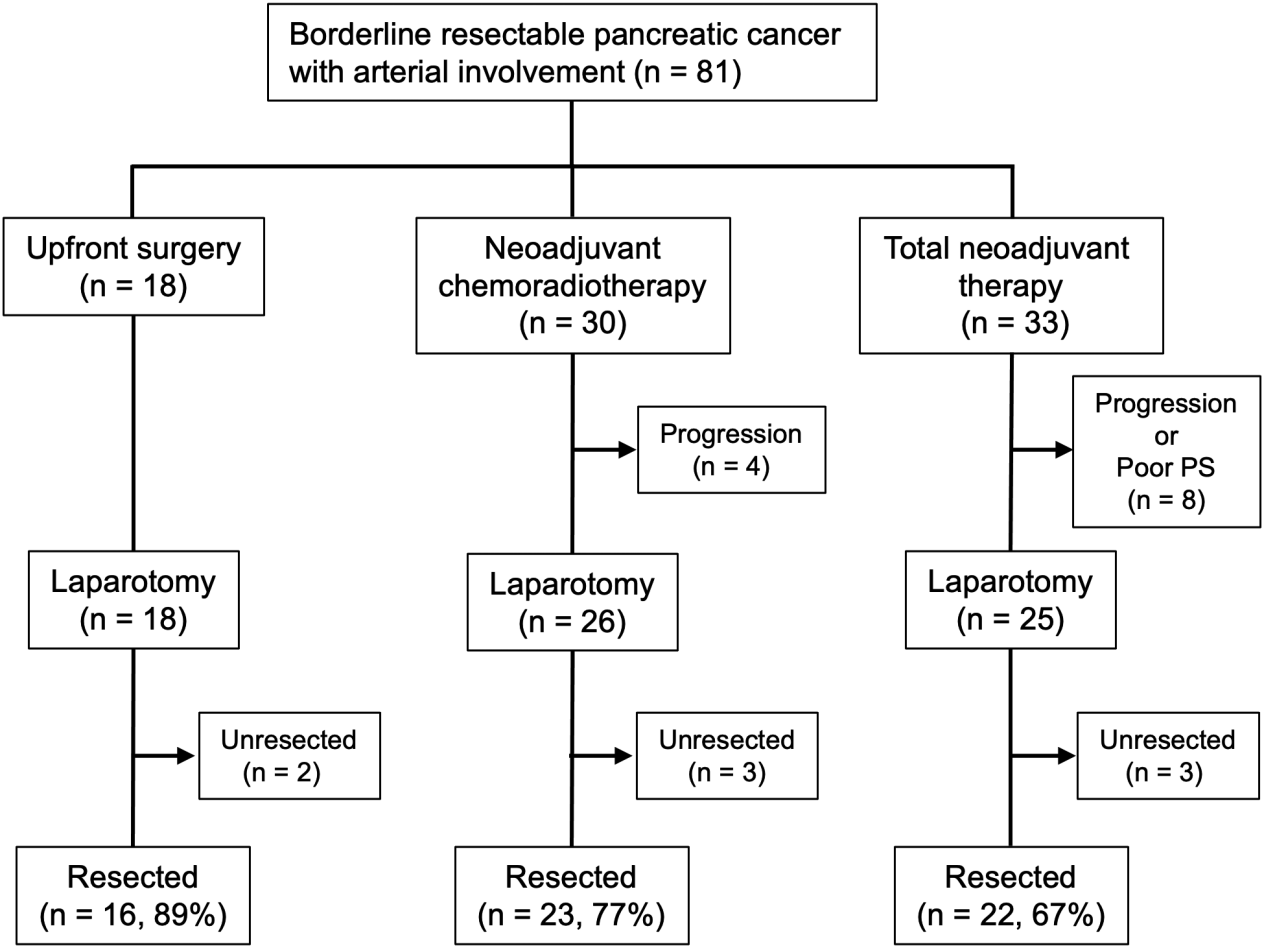
Study flowchart for 81 consecutive patients with BR‐A pancreatic cancer.

Among 81 patients with BR‐A pancreatic cancer, 18 patients underwent UFS, 30 patients underwent surgical resection after NACRT, and 33 patients were scheduled for TNT and received chemotherapy (Figure 2). Two patients in the UFS group and seven in the NACRT group did not undergo pancreatectomy because disease progression was confirmed at or before laparotomy. In the TNT group, 15 patients were initiated with the modified FOLFIRINOX regimen, 12 with GA, and six with GS. Multiple regimens were used in nine of 22 resected cases. There were no significant differences in clinical outcome between regimens in this study cohort. Eleven patients in the TNT group did not undergo pancreatectomy. Among these, 10 had progressive disease (lung metastasis in two, liver metastasis in two, local progression in three, and peritoneal dissemination in three patients). The other one patient did not undergo a pancreatectomy owing to their poor general health. The most common adverse events were neutropenia during the preoperative treatment. In the TNT group, two patients had adverse events requiring hospitalization (one with cholecystitis and one with diarrhea), while there was one patient with interstitial pneumonia in the NACRT group. The median follow‐up durations in all patients were 22.5 months (range; 2.0–111.4 months).

Table 1 summarizes the baseline characteristics of all the patients before any anticancer therapeutic intervention in the UFS, NACRT, and TNT groups. At the initial visit, there were no significant differences among the three groups, concerning age, sex, or carbohydrate antigen 19–9. The resection rates in the UFS, NACRT, and TNT groups were 89% (16/18), 77% (23/30), and 67% (22/33), respectively without a statistical difference (*p* = 0.208). The patient characteristics and postoperative outcomes of the 61 patients who underwent pancreatectomy in the UFS, NACRT, and TNT groups are revealed in Table 2. There were no significant differences in perioperative outcomes such as intraoperative blood loss, transfusion rate, and postoperative complications. In contrast, CA19‐9, preoperative tumor size, and histopathological effects, including pathological T and N factors, intraoperative cytology rates, and margin status rates, were significantly favorable for increasing the intensity of preoperative treatment. Notably, the rate of Tis, T1 (64%), N0 (86%), and negative margin status (95%) in the TNT group were extremely high compared with those in the other groups. S‐1 was frequently used as an adjuvant chemotherapy in the TNT group (72.7%). In addition, there was a significant difference in the completion rate of adjuvant chemotherapy after pancreatectomy between the groups. The highest percentage (68%) was observed in the TNT group. The most common reason for failure to complete adjuvant therapy was early recurrence during adjuvant therapy, which required the change of chemotherapy regimen.

**TABLE 1:** Characteristics of all 81 patients with borderline resectable pancreatic cancer with arterial involvement.

	UFS	NACRT	TNT	
	(*n* = 18)	(*n* = 30)	(*n* = 33)	*p*‐Value
Age (year)	64 (47–85)	70 (45–77)	69 (36–80)	0.691
Sex				
Male	10 (56)	21 (70)	22 (67)	0.584
Female	8 (44)	9 (30)	11 (33)	
Tumor location				
Head	13 (72)	15 (50)	21 (64)	0.279
Body and tail	5 (28)	15 (50)	12 (36)	
CA19‐9 at initial visit (IU/L)	176 (19–1776)	117 (2–2280)	162 (2–3134)	0.652
Tumor size (cm)	3.4 (1.9–4.7)	3.0 (1.5–6.0)	3.2 (1.2–8.0)	0.624
BMI (kg/m^2^)	20 (16–26)	22 (14–33)	22 (18–26)	0.069
PNI	48 (31–53)	49 (35–57)	48 (31–61)	0.437
Resection	16 (89)	23 (77)	22 (67)	0.208

*Note*: Values represent median (range), or number (%).

Abbreviations: BMI, body mass index; CA19‐9, carbohydrate antigen 19–9; NACRT, neoadjuvant chemoradiotherapy; PNI, prognostic nutrition index; TNT, total neoadjuvant therapy; UFS, upfront surgery.

**TABLE 2:** Characteristics of 61 resected patients with borderline resectable pancreatic cancer with arterial involvement.

	UFS	NACRT	TNT	*p‐*Value
	(*n* = 16)	(*n* = 23)	(*n* = 22)	
Age (year)	63 (47–83)	70 (47–76)	66 (56–78)	0.324
Sex				
Male	8 (50)	16 (70)	13 (59)	0.461
Female	8 (50)	7 (30)	9 (41)	
Tumor location				
Head	12 (75)	14 (61)	14 (64)	0.640
Body and tail	4 (25)	9 (39)	8 (36)	
CA19‐9 at initial visit (IU/L)	192 (19–1776)	99 (2–1135)	262 (3–3134)	0.029
Tumor size at initial visit (cm)	3.3 (1.9–4.7)	3.0 (1.5–4.6)	3.5 (1.2–8.0)	0.219
BMI (kg/m^2^)	20 (16–26)	22 (14–33)	23 (18–26)	0.132
PNI	48 (31–53)	49 (35–53)	48 (40–56)	0.542
CA19‐9 before surgery (IU/L)	192 (19–1776)	48 (1–356)	22 (3–89)	<0.001
Tumor size before surgery (cm)	3.3 (1.9–4.7)	2.6(1.0–4.5)	1.9 (1.0–8.0)	0.003
Blood loss (mL)	930 (40–3520)	590 (40–1305)	406 (50–2936)	0.142
Transfusion				
Yes	7 (44)	7 (30)	4 (18)	0.232
No	9 (56)	16 (70)	18 (82)	
Postoperative complications^a^				
Grade III–IV	3 (19)	7 (30)	4 (18)	0.557
Grade 0–II	13 (81)	16 (70)	18 (82)	
Pancreatic fistula (ISGPS)				
Grade B, C	3 (19)	5 (22)	2 (9)	0.497
Biochemical leak	13 (81)	18 (78)	20 (91)	
Pathological T stage				
Tis, T1	0 (0)	6 (26)	14 (64)	<0.001
T2	9 (56)	13 (57)	7 (32)	
T3, T4	7 (44)	4 (17)	1 (5)	
Pathological N stage				
0	3 (19)	16 (70)	19 (86)	<0.001
1	6 (38)	6 (26)	3 (14)	
2	7 (44)	1 (4)	0 (0)	
Positive washing cytology				
Yes	4 (25)	1 (4)	0 (0)	0.015
No	12 (75)	20 (96)	22 (100)	
Margin status				
R1	9 (56)	5 (22)	1 (5)	0.001
R0	7 (44)	18 (78)	21 (95)	
Evans classification				
I, II	–	22 (96)	15 (68)	0.012
III, IV	–	1 (4)	7 (32)	
Regimens of adjuvant therapy				
Gemcitabine	6 (38)	11 (48)	0 (0)	<0.001
S‐1	4 (25)	8 (35)	16 (73)	
WHF/GEM	3 (19)	4 (17)	6 (27)	
Completion of adjuvant therapy				
Yes	4 (25)	12 (52)	15 (68)	0.031
No	12 (75)	11 (48)	7 (32)	

*Note*: Values represent median (range), or number (%).

Abbreviations: BMI, body mass index; CA19‐9, carbohydrate antigen 19–9; ISGPS, International Study Group of Pancreatic Surgery; NACRT, neoadjuvant chemoradiotherapy; PNI, prognostic nutrition index; TNT, total neoadjuvant therapy; UFS, upfront surgery; WHF/GEM, weekly high‐dose 5‐fluorouracil/gemcitabine.

^a^ Clavien–Dindo classification.

### 3.2 Survival

**FIGURE 3:**
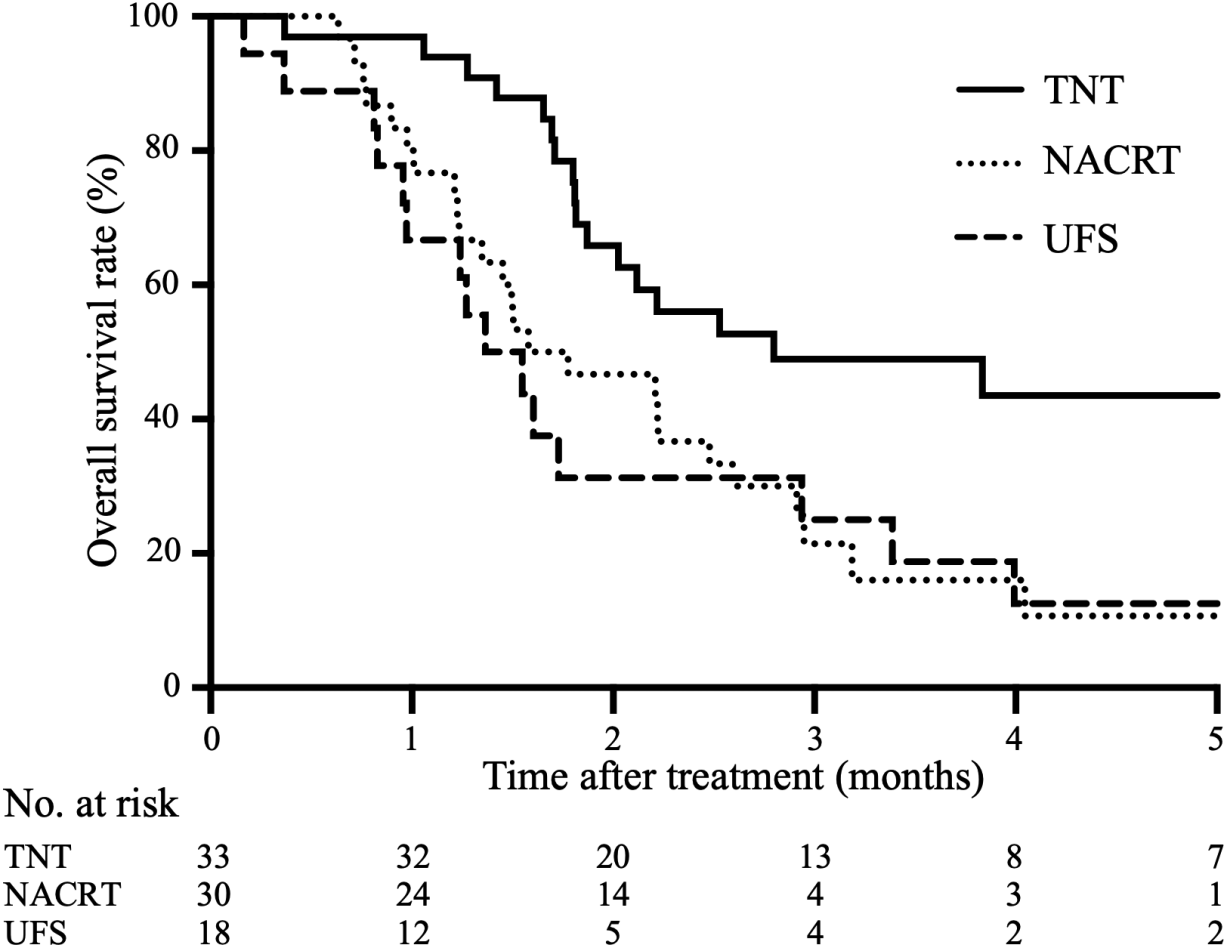
Kaplan–Meier analysis of overall survival in comparison among all the patients in total neoadjuvant therapy (TNT) groups, neoadjuvant chemoradiotherapy (NACRT), and upfront surgery (UFS). The prognosis of the TNT group was significantly better than that the UFS and NACRT groups (vs. UFS: *p* = 0.007, vs. NACRT: *p* = 0.016). There were no significant differences between the UFS and NACRT groups (*p* = 0.604).

We compared the prognoses of the patients, including those with unresected BR‐A, in each group (Figure 3). There were no significant differences in overall survival between the patients in the UFS and NACRT groups (*p* = 0.604). The median survival time (MST) of the patients in the UFS and NACRT groups were 16.6 and 19.2 months (M), respectively. These data clearly demonstrated that the preoperative treatment with conventional NACRT has no significant impact on the prognosis of the patients with BR‐A pancreatic cancer. In contrast, the prognosis of all the patients in the TNT group (MST, 33.5 M) was significantly greater than that of patients in the UFS and NACRT groups (*p* = 0.007 and *p* = 0.016, respectively).

**FIGURE 4:**
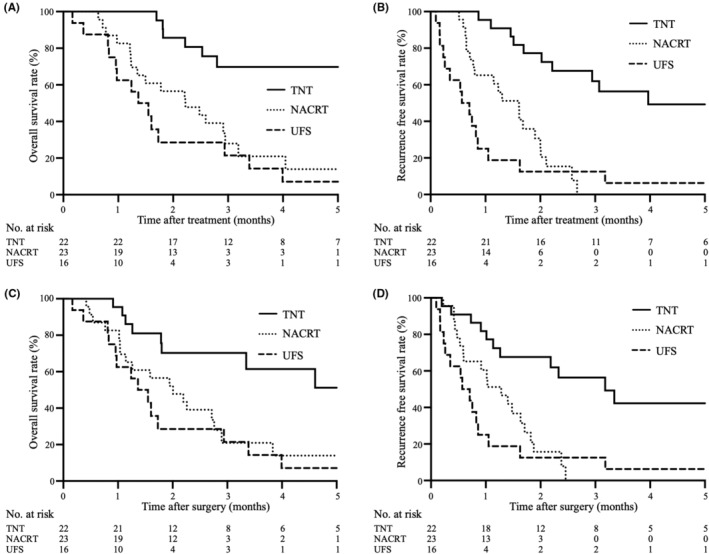
Kaplan–Meier analysis of overall survival (OS) and recurrence‐free survival (RFS) in comparison among the resected cases in the upfront surgery (UFS), neoadjuvant chemoradiotherapy (NACRT), and total neoadjuvant therapy (TNT) groups. (A) OS from the start of treatment of the TNT group was significantly better than that of the UFS and NACRT groups (vs. UFS: *p* < 0.001, vs. NACRT: *p* = 0.003). There were no significant differences between the UFS and NACRT group (*p* = 0.144). (B) RFS from the start on treatment of the TNT group was significantly better than that of the UFS and NACRT groups (vs. UFS: *p* < 0.001, vs. NACRT: *p* < 0.001). There were no significant differences between the UFS and NACRT groups (*p* = 0.223). (C) OS after surgery of the TNT group was significantly better than that of the UFS and NACRT groups (vs. UFS: *p* = 0.003, vs. NACRT: *p* = 0.015). There were no significant differences between the UFS and NACRT groups (*p* = 0.382). (D) RFS after surgery of the TNT group was significantly better than that of the UFS and NACRT groups (vs. UFS: *p* < 0.001, vs. NACRT: *p* < 0.001). There were no significant differences between the UFS and NACRT groups (*p* = 0.282).

Furthermore, we evaluated OS and RFS from the time of initial treatment only in the resected cases in the three groups (Figure 4A,B). The MST of the patients in the TNT group (75.1 M) was markedly better than that of the patients in the UFS and NACRT groups (16.6 M, *p* < 0.001 and 27.0 M, *p* = 0.003, respectively). The median RFS in the TNT groups (48.3 M) was significantly greater than that in the UFS and NACRT groups (6.9 M, *p* < 0.001 and 19.5 M, *p* < 0.001, respectively). There were no significant differences between the UFS and NACRT groups (*p* = 0.223). Next, we evaluated OS and RFS from the time of surgery (Figure 4C,D). The MST of the patients in the TNT group (92.5 M) was markedly better than that of the patients in the UFS and NACRT groups (16.6 M, *p* = 0.003 and 24.1 M, *p* = 0.015, respectively). The median RFS in the TNT groups (38.1 M) was also significantly greater than that in the UFS and NACRT groups (6.9 M, *p* < 0.001 and 15.4 M, *p* < 0.001, respectively). There were no significant differences between the UFS and NACRT groups (*p* = 0.282).

### 3.3 Recurrence site

Recurrences were observed in 15 patients (93.8%) in the UFS group, in 20 patients (87.0%) in the NACRT group, and in 10 patients (45.5%) in the TNT group. Local recurrence was observed in four patients (25%) in the UFS group, two (8.7%) in the NACRT group, and none in the TNT group. Recurrence to multiple sites was observed in four patients (25%) in the UFS group, five (21.7%) in the NACRT group, and one (4.5%) in the TNT group.

### 3.4 Prognostic factors

We analyzed the prognostic factors of 81 patients with BR‐A pancreatic cancer (Table 3). Univariate analysis indicated that TNT (hazard ratio [HR]:0.46, 95% confidence interval [CI]: 0.27–0.79, *p* = 0.005) and surgical resection (HR: 0.40, 95% CI: 0.23–0.69, *p* = 0.001) were significantly associated with overall survival. Furthermore, the multivariable analysis revealed both TNT (HR: 0.44, 95% CI: 0.26–0.75, *p* = 0.002) and surgical resection (HR: 0.38, 95% CI: 0.22–0.65, *p* < 0.001) were also the independent prognostic factors in overall survival for the patients with BR‐A. Next, we analyzed the prognostic factors in 61 resected cases (Table 4). Univariate analysis indicated that normalization of CA19‐9 before surgery (HR: 0.35, 95% CI: 0.18–0.65, *p* < 0.001), TNT (HR: 0.27, 95% CI: 0.13–0.58, *p* < 0.001), no lymph node metastasis (HR: 0.32, 95% CI: 0.17–0.60, *p* < 0.001), R0 (HR: 0.42, 95% CI: 0.22–0.80, *p* = 0.009), and completion of adjuvant chemotherapy (HR: 0.38, 95% CI: 0.10–0.38, *p* < 0.001) were significantly associated with overall survival. Furthermore, the multivariable analysis revealed no lymph node metastasis (HR: 0.30, 95% CI: 0.13–0.67, *p* = 0.003) and completion of adjuvant chemotherapy (HR: 0.15, 95% CI: 0.07–0.31, *p* < 0.001) were also the independent prognostic factors in overall survival for the resected cases with BR‐A.

**TABLE 3:** Univariate and multivariate Cox hazard regression analysis for overall survival of borderline resectable pancreatic cancer with arterial involvement.

Factors	No. of patients	Univariate analysis		Multivariate analysis	
	*n* = 81	HR (95% CI)	*p‐*Value	HR (95% CI)	*p‐*Value
Age, years					
<69	41	0.95 (0.57–1.56)	0.830		
≥69	40	Ref.			
Sex					
Male	53	1.12 (0.65–1.93)	0.676		
Female	28	Ref.			
Tumor location					
Head	49	0.85 (0.51–1.43)	0.545		
Body and tail	32	Ref.			
CA19‐9 at initial visit (IU/L)					
≤37	15	0.72 (0.36–1.65)	0.350		
>37	66	Ref.			
CA19‐9 at initial visit (IU/L)					
≤131	41	1.15 (0.70–1.90)	0.584		
>131	40	Ref.			
Tumor size (cm)					
≤3	40	0.97 (0.59–1.59)	0.894		
>3	41	Ref.			
BMI (kg/m^2^)					
<22	42	1.18 (0.72–1.95)	0.514		
≥22	39	Ref.			
PNI					
<40	7	1.85 (0.81–4.22)	0.142		
≥40	74	Ref.			
NACRT					
Yes	30	1.47 (0.88–2.45)	0.144		
No	51	Ref.			
TNT					
Yes	33	0.46 (0.27–0.79)	0.005	0.44 (0.26–0.75)	0.002
No	48	Ref.			
Resection					
Yes	61	0.40 (0.23–0.69)	0.001	0.38 (0.22–0.65)	<0.001
No	20	Ref.			

Abbreviations: BMI, body mass index; CA19‐9, Carbohydrate antigen 19–9; CI, confidence interval; HR, hazard ratio; NACRT, neoadjuvant chemoradiotherapy; PNI, prognostic nutrition index; Ref, reference; TNT, total neoadjuvant therapy.

**TABLE 4:** Univariate and multivariate Cox hazard regression analysis for overall survival of resected cases of borderline resectable pancreatic cancer with arterial involvement.

Factors	No. of patients	Univariate analysis		Multivariate analysis	
	*n* = 61	HR (95% CI)	*p‐*Value	HR (95% CI)	*p‐*Value
Age, years					
<69	31	0.74 (0.40–1.37)	0.336		
≥69	30	Ref.			
Sex					
Male	37	1.21 (0.64–2.28)	0.552		
Female	24	Ref.			
CA19‐9 at initial visit (IU/L)					
≤37	10	0.62 (0.24–1.57)	0.311		
>37	51	Ref.			
CA19‐9 at initial visit (IU/L)					
≤163	31	0.74 (0.39–1.41)	0.364		
>163	30	Ref.			
Tumor size at initial visit (cm)					
≤3	31	1.07 (0.58–1.96)	0.836		
>3	30	Ref.			
CA19‐9 before surgery (IU/L)					
≤37	32	0.35 (0.18–0.65)	<0.001	0.86 (0.37–2.02)	0.731
>37	29	Ref.			
Tumor size before surgery (cm)					
≤2.5	31	0.68 (0.37–1.25)	0.211		
>2.5	30	Ref.			
NACRT					
Yes	23	1.59 (0.86–2.97)	0.141		
No	38	Ref.			
TNT					
Yes	22	0.27 (0.13–0.58)	<0.001	0.37 (0.13–1.06)	0.063
No	39	Ref.			
Lymph node metastasis					
No	38	0.32 (0.17–0.60)	<0.001	0.30 (0.13–0.67)	0.003
Yes	23	Ref.			
R status					
R0	46	0.42 (0.22–0.80)	0.009	0.71 (0.33–1.53)	0.380
R1	15	Ref.			
Completion of AC					
Yes	31	0.19 (0.10–0.38)	<0.001	0.15 (0.07–0.31)	<0.001
No	30	Ref.			

Abbreviations: AC, adjuvant chemotherapy; BMI, body mass index; CA19‐9, Carbohydrate antigen 19–9; CI, confidence interval; HR, hazard ratio; NACRT, neoadjuvant chemoradiotherapy; PNI, prognostic nutrition index; Ref, reference; TNT, total neoadjuvant therapy.

## 4 DISCUSSION

Although neoadjuvant therapy for patients with BRPC has been recommended in recent guidelines, the optimal regimens and prognostic impact have not yet been fully elucidated. Recently, TNT has been proposed as a new preoperative strategy for patients with locally advanced pancreatic cancer.^12^, ^13^, ^14^, ^22^ Although TNT for pancreatic cancer has not been completely defined, it is usually considered to be a combined preoperative treatment with intense induction of chemotherapy followed by CRT. Although the indication, regimen, and treatment period of TNT varied, several studies have reported favorable outcomes for patients with locally advanced pancreatic cancer.^12^, ^14^, ^23^ However, most studies analyzed data together on unresectable locally advanced and borderline resectable pancreatic cancer in the same study cohort. Therefore, it remains ambiguous whether any resectablility categories (i.e., BR‐PV, BR‐A, or URLA) are truly suitable for TNT.

In our previous study to determine the optimal indication of NACRT for borderline resectable pancreatic cancer, we established those patients treated with NACRT had a better prognosis than those without NACRT in the BR‐PV group. However, it differed from the BR‐A group.^9^ The results indicated that NACRT was insufficient for achieving satisfactory therapeutic effects for BR‐A pancreatic cancer. Based on these data, TNT has been used in patients with BR‐A since 2014. As a result, the survival of patients with BR‐A has markedly improved, especially in resected cases. On the other hand, some institutions have adapted gemcitabine plus S‐1 based NACRT regimens for BR‐A pancreatic cancer. Yabushita et al. reported a prospective study about gemcitabine plus S‐1‐based NACRT regimen for BR‐A pancreatic cancer.^24^ Although direct comparison is not optimal, considering the resection rate of 74% and the MST of 16.4 months in their report, our results may be favorable. However, well‐designed clinical trials are needed to answer this critical clinical question about the optimal regimen as well as duration.

In general, prolonged preoperative treatment has advantages and disadvantages. Sufficient neoadjuvant treatment with a longer duration of use may allow surgeons to select patients who are more suitable for resection after adequate disease control. On the other hand, if nonsurgical treatment is ineffective, the surgeon may miss the opportunity to perform a curative resection and the patient may also miss the chance for cure. Therefore, the duration of treatment in patients with BRPC is a critical clinical question. To date, there are little data and no evidence regarding the proper period for TNT. Reports by Satoi et al. reveal important indicators of treatment methods.^25^ Although the results of the study were initially on unresectable pancreatic cancer and not on BRPC, they suggested that there was a preferable period of treatment. Based on our data analysis of BR‐A in 2014, we administrated intense chemotherapy for 6 months, followed by 2 months of CRT before surgery. The resection rate (71%) of the TNT group was comparable with that of the NACRT group and better than that (52%) in patients with BRPC treated with preoperative chemoradiotherapy in a randomized phase III trial (PREOPANC).^5^ Importantly, the MST at 46 months in the TNT group was much better than that in the NACRT groups and that of BRPC patients in the PREOPANC study. Therefore, the preoperative period may be appropriate for our treatment regimen. However, the indication for surgery for BRPC remains subjective and non‐scientific. The application of emerging precision‐medicine may be needed for better treatment.^26^, ^27^, ^28^ The development of novel biomarkers may provide valuable sources to decide when surgical resection should be performed and discriminate between those who will benefit from TNT and those who will not.

The role of chemoradiotherapy as a neoadjuvant therapeutic strategy for pancreatic cancer remains unclear. Jang et al. reported a significant survival benefit of NACRT in patients with BRPC compared with up‐front surgery in a phase II/III study.^29^ The results may not provide firm evidence due to the relatively small number of the study cohort. Furthermore, a well‐designed study, that is, PROPANC, on preoperative CRT for resectable and BRPC patients did not meet a primary end point.^5^ Although the prognosis of the BRPC patients with CRT in subgroup analysis was significantly better than those without CRT, the MST of 17.6 months in the CRT group seems insufficient. However, the factors related to local control, such as the rates of pathologic lymph node metastasis and R0 resection were significantly better than those without CRT. Similarly, our previous study revealed that NACRT brought better local control compared with upfront surgery for BR‐A, however, it did not improve prognosis.^9^ In contrast, TNT which included the same CRT regimen showed a significantly better prognosis than the NACRT group. This may indicate that chemotherapy before CRT effectively controlled occult systemic disease, which could not be confirmed in the imaging study. However, it is still unknown whether long‐term systemic chemotherapy or TNT including CRT is the better treatment for BR‐A. Therefore, further studies are required to confirm the necessity of CRT for BRPC.

This study has certain limitation. First, because this study adopted a retrospective design, patient as well as treatment selection bias may have affected the results and interpretations. Second, the study cohort included only a limited number of patients, making it difficult to reach a definitive conclusion. However, a comparison of the three cohorts provided important consecutive information regarding the treatment of patients with BR‐A pancreatic cancer. Finally, chemotherapy regimens before CRT were selected at the discretion of the treating physicians. Taken together, well‐designed clinical studies are needed to draw definitive conclusions. Nonetheless, the present study provides important information regarding the prognostic impact of TNT in patients with BR‐A.

## 5 CONCLUSIONS

The total neoadjuvant strategy for the patients with BR‐A markedly improved the prognosis compared with upfront surgery or conventional NACRT. Although clinical questions regarding TNT remain unresolved, further prospective trials are warranted to provide better care for patients with potentially fatal diseases.

## AUTHOR CONTRIBUTIONS

Study concepts: T. Akahori, T. Terai. Project development: M. Sho, S. M. Nagai, K. Nakamura. Data acquisition: Y. Kohara, S. Yasuda, Y. Matsuo, S. Doi, T. Sakata. Analysis and interpretation of data: T. Akahori, T. Terai.

## FUNDING INFORMATION

No funding was received for this study.

## CONFLICT OF INTEREST STATEMENT

Masayuki Sho is an editorial board member of *Annals of Gastroenterological Surgery*.

## ETHICS STATEMENTS

Approval of the research protocol: This research was approved by the Ethics Committee of the institution (Committee of Nara Medical University, Approval Number 2383) and it conforms to the provisions of the Declaration of Helsinki.

Informed Consent: Because this was a retrospective review of medical records, consent to participate was not required from the patients.

Registry and the Registration No. of the study/trial: N/A.

Animal Studies: N/A.
